# Comparable Efficacy in Ischemic and Non-Ischemic ICD Recipients for the Primary Prevention of Sudden Cardiac Death

**DOI:** 10.3390/biomedicines9111595

**Published:** 2021-11-02

**Authors:** Andreea Maria Ursaru, Antoniu Octavian Petris, Irina Iuliana Costache, Nicolae Dan Tesloianu

**Affiliations:** 1 Department of Cardiology, Emergency Clinical Hospital “Sf. Spiridon”, 700111 Iași, Romania; andreea_ursaru@yahoo.com (A.M.U.); antoniu.petris@yahoo.ro (A.O.P.); dan_tesloianu@yahoo.com (N.D.T.); 2 Department of Cardiology, “Grigore. T. Popa” University of Medicine and Pharmacy, 700111 Iași, Romania

**Keywords:** primary prevention of sudden cardiac death, non-ischemic cardiomyopathy, ischemic cardiomyopathy, appropriate ICD therapy, mortality rate comparison

## Abstract

(1) Background: In patients suffering from heart failure, the main causes of death are either hemodynamic failure, or ventricular arrhythmias. The only tool to significantly reduce arrhythmic sudden death is the implantable cardioverter defibrillator (ICD), but not all patients benefit to the same extent from these devices. (2) Methods: The primary outcome of this single-center study was defined as cardiovascular death in patients with ischemic and non-ischemic heart failure who have benefited from ICD therapy. The secondary outcomes were death from any cause, sudden cardiac death, ICD-related therapies (appropriate antitachycardia pacing or shock therapy for ventricular tachycardia or fibrillation) and recurrences of ventricular tachyarrhythmias. (3) Results: A total of 403 consecutive ICD recipients—symptomatic heart failure patients with ICD for the primary prevention of sudden cardiac death—were included retrospectively: 59% ischemic cardiomyopathy (ICMP) and 41% non-ischemic cardiomyopathy (NICMP) patients. Within a median follow-up period of 36 months, the incidence of cardiovascular mortality was not significantly different in patients with NICMP and ICMP: the primary outcome had occurred in 9 patients (5.4%) in the NICMP group and in 14 patients (5.9%) in the ICMP group (hazard ratio 1; 95% confidence interval (CI) 0.45 to 2.28; *p* = 0.97). All-cause mortality occurred in 14 of 166 patients (8.4%) in the NICMP group and 18 of 237 patients (7.6%) in the ICMP group. Sudden cardiac death occurred in two patients (1.2%) in the NICMP group and in four patients (1.7%) in the ICMP group (hazard ratio 0.71; 95% CI, 0.13 to 3.88; *p* = 0.69). The rate of appropriate device therapies was comparable in both groups. (4) Conclusions: In this study, ICD implantation for primary prevention of sudden cardiac death in patients with symptomatic systolic heart failure was associated with similar rates of cardiovascular and all-cause mortality in patients with ischemic heart disease, and in patients with heart failure from other causes. NICMP and ICMP showed comparable rates of recurrent ventricular tachyarrhythmias and appropriate ICD therapies.

## 1. Introduction

In patients with heart failure and reduced left ventricular ejection fraction (LVEF < 35%), there is an increased risk of sudden cardiac death (SCD) due to ventricular arrhythmias, with the highest risk in those who survived an episode of ventricular fibrillation (VF) or sustained ventricular tachycardia (VT) [1]. In subjects with secondary prevention of SCD, where no reversible cause such as an acute myocardial infarction can be identified, an implantable cardioverter defibrillator (ICD) is recommended with a class IA indication according to European Society of Cardiology (ESC) guidelines [2,3]. The situation is more complex in patients with ICD therapy for the primary prevention of SCD. In the new 2021 ESC Heart Failure Guidelines, primary prevention of SCD in patients with symptomatic systolic non-ischemic heart failure was downgraded from a 1B recommendation class in the 2016 ESC Heart Failure Guidelines, to a IIaB recommendation, as opposed to the class 1A recommendation for patients with ischemic heart failure, which has remained a constant indication [2,3]. In the American Heart Association Guidelines, on the other hand, ICD implantation for primary prevention of SCD in patients with symptomatic systolic heart failure is a class 1A recommendation, with no differentiation between patients with ischemic and non-ischemic cardiomyopathy [4]. This difference arises from the trials on which the guidelines are based: the American Heart Association Guidelines refer to the Sudden Cardiac Death in Heart Failure Trial (SCD-HeFT), the 2016 European Guidelines consider the Defibrillators in Non-Ischemic Cardiomyopathy Treatment Evaluation (DEFINITE), while the latest ESC Guidelines version grounds its recommendations on the DANISH study. The SCD-HeFT trial proved the benefit of ICD implantation in patients with non-ischemic heart failure with regard to all-cause mortality [5]. With opposite results, the more recent randomized Danish Study to Assess the Efficacy of ICDs in Patients with Non-ischemic Systolic Heart Failure on Mortality (DANISH) trial demonstrated a significant reduction in SCD in patients with LVEF ≤ 35%, but without a significantly lower long-term rate of death from any cause when compared to usual clinical care [6]. Subgroup analyses of the DANISH trial have, however, shown contrasting results: in patients under 70 years of age or in those with less severe heart failure, reduction in all-cause mortality was demonstrated [7]. Also contrary to the DANISH trial results, there are meta-analyses showing a significant survival benefit due to the implantation of ICD in non-ischemic cardiomyopathy (NICMP) patients [8,9,10].

Considering the available literature data, it is justifiable to ask whether we need to rethink the indication for ICD therapy in primary prevention in the entire non-ischemic population. In the current study, we aim to demonstrate that NICMP patients have a similar percentage of reduction in arrhythmogenic deaths and all-cause mortality (cardiovascular death and non-cardiovascular death) when compared with patients in the ischemic cardiomyopathy group.

## 2. Materials and Methods

### 2.1. Study Design and Patients

This retrospective study was conducted at a single academic center in a consecutive series of patients. It included patients with “de novo” ICD implants between January 2017 and January 2021, with a median follow-up time of 36 months (range, 7 months to 55 months). Patients of either sex who were more than 18 years of age (there was no upper age limit) with clinical heart failure and a left ventricular ejection fraction equal to or below 35% despite optimal medical therapy were included. The New York Heart Association (NYHA) functional classes II or III represented inclusion criteria for ICD recipients, and NYHA class IV for CRT recipients. Patients with previous conventional pacemakers and CRT-P were also included. Permanent atrial fibrillation with no upper rate limit and end-stage renal failure (dialysis) were not exclusion criteria, in contrast to the majority of the previous studies. Exclusion criteria were defined as follows: patients on the urgent waiting list for a heart transplant, uncorrected congenital heart disease, obstructive cardiomyopathy, active myocarditis, constrictive pericarditis, human immunodeficiency virus (HIV)-positive patients with an expected survival of less than 3 years due to HIV, recent history of alcohol or illicit drug abuse disorder (within 3 months), lack of informed consent, age under 18 years, and severe depression or other major psychiatric illness.

The patients were divided into 2 groups: patients with ischemic cardiomyopathy (ICMP) and patients with NICMP. For the ICMP group, patients with a history of previous myocardial infarction documented by the finding of an abnormal Q wave on electrocardiography, elevated cardiac-enzyme levels on laboratory testing during hospitalization for acute coronary syndrome, localized akinesia on echocardiography, with evidence of obstructive coronary disease on angiography, and an ejection fraction of 35% or less within three months before entry, as assessed by angiography or echocardiography, were included. In patients with non-ischemic systolic heart failure with LVEF ≤ 35%, the exclusion of myocardial ischemia was done by coronary angiography (the majority of patients) and computed tomography angiography. All patients were primarily in NYHA functional class II, III, or ambulatory class IV. Patients with ICD therapy indication, NYHA class II or III, and a native QRS complex greater than or equal to 150 milliseconds were implanted with a CRT-D. NYHA functional class IV patients received cardiac resynchronization therapy with defibrillation therapy (CRT-D), the cutoff value for the duration of the native QRS complex being 130 milliseconds.

Cardiovascular deaths were subclassified as sudden or non-sudden. Sudden cardiac deaths were defined by World Health Organization criteria, for which no obvious non-arrhythmic cause of death was found, including death occurring unexpectedly in a previously stable patient, sudden unexpected death within 1 h of acute symptom onset or worsening of symptoms (if witnessed), or within 24 h of the last observation at baseline (if unwitnessed), such as when the patient was found in bed [11]. Sudden deaths were further evaluated through device interrogation to determine whether a device concern was present (hardware failures, device algorithm issues, device programming issues). This was not the case in either of the deceased. Non-cardiovascular deaths were defined as all deaths not adjudicated as cardiovascular death. Cardiovascular deaths classified as non-sudden and all non-cardiovascular deaths were categorized together as non-sudden deaths.

### 2.2. ICD Therapy

ICD therapy was selected to consist of antitachycardia pacing (ATP) therapies and shocks. Single and dual-chamber ICDs and biventricular devices were implanted. The defibrillation leads were single-coil leads. The goal was to treat only rapid, sustained ventricular tachycardia or ventricular fibrillation, and to minimize excessive interventions, so the devices were uniformly programmed according to the MADIT-RIT delayed therapy arm (170–199 bmp with 60 s delay; 200–249 bmp with 12 s delay; >=250 bpm with 2.5 s delay) and the ADVANCE III trial, with longer delay—30 of 40 instead of the conventional 18 of 24. A “monitor only” ventricular tachycardia detection interval was set at 150 bpm for all patients [12,13]. Because of the potential of pacing to worsen CHF, the minimal pacing rate was set to 40 beats per minute. No rate-responsive pacing was allowed [14,15,16].

In general, two or three therapy zones (mainly one VT zone, one VF zone, and possibly an additional fast VT (FVT) zone) were programmed. VT was primarily treated with ATP and possibly consecutive ICD shocks. VF was primarily treated with ICD shock with ATP during charging. Over time, changes in programming routines have occurred, consisting of further prolongation of the tachycardia duration criteria or an increase of cut-off rates in detection zones, in order to avoid repetitive inappropriate shocks.

Appropriate therapy was defined as shock or ATP for real VT or VF following analysis of the intracardiac electrograms.

### 2.3. Statistical Analysis

The cumulative survival plots were estimated according to the Kaplan–Meier method. Survival in groups was compared with the log rank test. Univariate Cox regression analysis was performed to identify significant independent predictors of outcome. Results are reported as the adjusted hazard ratio (HR) with a 95% confidence interval (CI). A two-sided *p*-value < 0.05 was considered statistically significant. Statistical analyses were performed using the Survival R package, version 3.2-3.

### 2.4. Drug Therapy and Follow-Up

Follow-up was performed at 1 month and 3 months after discharge, and then every six months. The visits consisted of clinical and paraclinical examinations, including interrogation of the devices. Clinical surveillance involved monitoring of the patients and anticipated visits in case of worsening of the clinical status and occurrence of symptoms, including internal electrical shocks. The medication and, where necessary, reprogramming of the device were adapted.

The patients were receiving chronic optimal medical therapy (Table 1), including novel drug therapy for heart failure (Angiotensin Receptor-Neprilysin Inhibitor, sodium-glucose cotransporter-2 inhibitors).

## 3. Results

Baseline characteristics, demographic, clinical, and paraclinical data of the patients with ischemic and non-ischemic cardiomyopathy are presented in Table 1. A total of 403 patients, with a median follow-up time of 36 months, were divided into two groups: 166 patients in the NICMP group and 237 patients in the ICMP group, with no significant differences regarding baseline characteristics noticeable between the two groups (*p* > 0.05). The median age of the study population was 65 years (range, 19–83 years). The great majority of the subjects received heart failure drug therapy available at the time of implant, in accordance with the ESC guidelines; 19% of the patients in NICMP group (32 patients) and 17% (40 patients) in the ICMP group received CRT-D. Amiodarone was initiated in a significant number of patients (55 patients—33% in NICMP group, respectively 97 patients—41% in ICMP group), in order to avoid unnecessary shocks.

Table 2 shows the incidence of death from any cause, cardiovascular death, with subgroups: sudden cardiac death and other cardiovascular death (represented by congestive heart failure and fatal myocardial infarction), non-cardiovascular death, non-sudden deaths, appropriate, and inappropriate ICD therapy.

The primary outcome, cardiovascular death, occurred in nine patients (5.4%) in the NICPM group, and in 14 patients (5.9%) in ICPM group. The hazard ratio for cardiovascular death in the NICMP group as compared with the ICMP group was 1 (95% confidence interval (CI) 0.45 to 2.28; *p* = 0.97).

Death from any cause occurred in 14 patients (8.4%) in the NICMP group and in 18 patients (7.6%) in the ICMP group (hazard ratio, 1.1; 95% CI, 0.54. to 2.21 *p* = 0.78). Sudden cardiac death occurred in two patients (1.2%) in the NICMP group and in four patients (1.7%) in the ICMP group (hazard ratio, 0.71; 95% CI, 0.13 to 3.88; *p* = 0.69). Non-sudden cardiovascular death occurred in seven patients (4.2%) in the NICMP group and in 10 patients (4.2%) in the ICMP group (hazard ratio, 1.1; 95% CI, 0.44 to 2.87; *p* = 0.79). The clinical outcome of non-sustained ventricular tachycardia/ventricular fibrillation was registered with similar frequency in the two groups: 130 patients (78.3%) in the NICMP group and in 192 patients (81%) in the ICMP group (hazard ratio, 0.68; 95% CI, 0.94 to 1.1; *p* = 0.9); termination of ventricular tachyarrhythmia by antitachycardia pacing or/and appropriate shock was observed in 16 patients in the NICMP group (9.6%), and 24 patients (10.1%) in the ICMP group (hazard ratio, 1.2; 95% CI, 0.62 to 2.03; *p* = 0.8). Inappropriate shocks were all due to atrial fibrillation with rapid ventricular conduction, with the exception of one, due to electromagnetic interference (4.2% in NICMP group and 3% in ICMP group—hazard ratio, 0.72; 95% CI, 0.42 to 2.93; *p* = 0.52). Serious complications related to defibrillator therapy were infrequent. Device infection requiring lead extraction was present in four patients. No deaths occurred during implantation.

## 4. Discussion

The present study proves comparable results of ICD implantation for primary prevention of SCD in patients with ischemic and non-ischemic patients, the primary endpoint of cardiovascular mortality (subdivided into sudden cardiac death and other causes of cardiovascular death) having similar percentages in the two groups: 5.4% in the NICMP group and 5.9% in the ICMP group. In comparison with DANISH trial, which reported a 13.8% cardiovascular mortality in the ICD group, the incidence of cardiovascular death in our study is lower. Our analysis also showed comparable rates when evaluating the prognostic impact of cardiomyopathy type (NICMP versus ICMP) on the secondary endpoints, including death from any cause, sudden cardiac death, ICD-related therapies (ATP or shock therapy for ventricular tachycardia or fibrillation), and recurrences of ventricular tachyarrhythmias. The overall cumulative incidence of all-cause mortality of the total study population was 8.4% in NICMP patients and 7.6% in ICMP patients. Compared with the landmark trials, the cumulative incidence off all-cause mortality was relatively low: SCD-HeFT found a total mortality rate of 22% in the ICD group, while DANISH reported that death from any cause occurred in 21.6% of the ICD group. The overall mortality rate at two years in the DEFINITE trial was 7.9% in the ICD group. Sudden cardiac death occurred in 1.2% and 1.7% patients in our study when compared with 4.3% in the ICD group of the DANISH study. The rate of appropriate device therapies was similar in both groups of the present study, appropriate shock or ATP was present in 9.6% of the non-ischemic patients and 10.1% of the ischemic patients, while the DANISH trial reported termination of ventricular tachycardia by ATP in 17.4% of the ICD patients and appropriate shock for 11.5%.

There are three main studies that preponderantly influenced the practice in ICD implant in patients with primary prevention and NIMCP over time. The SCD-HeFT trial included 2521 patients with New York Heart Association (NYHA) class II or III congestive heart failure and a LVEF of 35% or less that were randomly assigned to placebo (847), amiodarone (845), and ICD therapy (829) patients. The study included both ischemic and non-ischemic subjects; 52% of the patients had ischemic cardiomyopathy and 48% non-ischemic cardiomyopathy, and the median follow-up time was 45.5 months. The trial proved that shock-only ICD therapy reduces overall mortality by 23 percent irrespective of the heart failure etiology, whereas amiodarone had no favorable effect on survival [5,17].

Given the well-documented benefit of ICD in patients with symptomatic heart failure caused by coronary artery disease [5,18], the DANISH trial included patients with symptomatic systolic heart failure not caused by coronary artery disease: 556 assigned to ICD implant and 560 assigned to usual clinical care. In contrast with the results of the present analysis, the DANISH study found that implantation of an ICD in patients with NICMP did not provide an overall survival benefit, although the risk of sudden cardiac death was halved [6]. However, a post hoc analysis of the study revealed that ICD implantation was associated with reduced all-cause mortality in patients ≤70 years of age, and that the benefit of ICD implantation decreased with older age, considering the fact that older patients were more likely to die of causes other than sudden cardiac death compared with younger patients, which might be a reason for the diminishing association between ICD implantation and all-cause mortality with advancing age [19,20].

The contrasting results between the DANISH trial and the present analysis might be explained by several differences. The DANISH study included a large number of CRT recipients (CRT was implanted in 58% of the patients and in 68% of patients >70 years of age) when compared to only 19% of the patients with NICMP which received CRT in our study. As an indication for CRT, we chose to include only patients with Class I and IIa indication in ESC guidelines and a native QRS complex duration greater than or equal to 150 milliseconds for patients with NYHA functional class II and III, while for patients with ambulatory class IV NYHA, a native QRS complex duration greater than or equal to 130 milliseconds was accepted. As is well known, patients with non-ischemic cardiomyopathy have shown higher response rates to CRT compared with patients with ischemic cardiomyopathy, differences explained partly by the myocardial substrate. CRT improves heart failure symptoms and prognosis and induces left ventricle reverse remodeling and increases its systolic function, but it has also been demonstrated that it reduces the rate of onset of new ventricular arrhythmias detected by ICDs in patients without a history of prior ventricular arrhythmias, an effect that was not observed in the subjects implanted for secondary prophylaxis [21,22,23]. The large proportion of patients with CRT in the DANISH trial, which may have lowered the overall mortality by disease modification, diminishes the chance of observing any effect of ICD on top of CRT, so this may be considered an a priori limitation of the study concerning ICD in NICMP.

Another difference between the two studies worthy of mention is the discrepancy of the analyzed groups: unlike the DANISH study, which compared non-ischemic heart failure patients assigned to ICD implant with non-ischemic heart failure patients assigned only to drug therapy, we analyzed ICMP and NICMP patients, with both groups assigned an ICD implant.

A significant distinction resides in the severity of the disease: the population of the DANISH study consisted predominantly of outpatients who were in stable condition, while the patients in our study were patients with severe symptomatic heart failure, including ambulatory Class IV NYHA (CRT-recipients), with a median NT-proBNP of 1698 pg/mL.

The DEFINITE trial is an older study that influenced the guidelines recommendations: 458 patients with NICMP, LVEF of less than 36%, and premature ventricular complexes or non-sustained VT were enrolled, of which 229 patients were randomly assigned to receive standard medical therapy, and 229 were assigned to standard medical therapy plus a single-chamber ICD. In patients with severe NICMP, the implantation of a defibrillator significantly reduced the risk of sudden death from arrhythmia, and reduced the risk of death from any cause to an extent that approached, but did not reach statistical significance. Patients were followed for a mean of 29 months. As in the case of the DANISH trial, the DEFINITE trial compared ICD patients with non-ICD patients; 25.3% of the ICD recipients were asymptomatic patients (NYHA class I), a group of patients for which there are currently no controlled, randomized studies demonstrating the value of an ICD, regardless of the etiology of the cardiomyopathy [24].

Summarizing, both of the above-mentioned studies, which apparently laid the groundwork against ICD therapy in NICMP, found a reduction in sudden death from arrhythmia and a benefit of ICD in reducing death of all causes in subgroup analyses. Moreover, the affiliation to the newer evidence in programming added to the evolution of device technology, which has resulted in greater patient safety and fewer complications. The newer-generation devices, with antitachycardia pacing (ATP was not available in the DEFINITE trial) and low-energy shocking capabilities for treatment of VT are all factors that lead to different outcomes in our study when compared with older trials like the DEFINITE and DANISH, besides the differences between the groups that were compared.

It is acknowledged that the effect of ICD therapy in patients with chronic heart failure may differ substantially depending on the programming of the device and the concomitant medication. Considering the potential harm from inappropriate shocks and the realization that long episodes of VT can self-terminate, a strategy of long detection was adopted in the present study. Our event rate of ICD therapy was lower than observed in older studies, which reflects the new programming, with increasing intervals to detect (high-rate detection and delayed therapy) and the fact that our study population were treated medically in accordance with the guidelines, with almost every patient receiving beta-blockers, inhibitors of the renin–angiotensin system, and mineralocorticoid-receptor antagonists. Just under a quarter of the patients in both groups received also new drugs for heart failure: ARNI (angiotensin receptor neprilysin inhibitor) and sodium-glucose cotransporter-2 (SGLT2) inhibitors. A consistent segment of our population, representing the majority of patients with NSVT, received amiodarone. Although SCD-HeFT demonstrated the superiority of ICD therapy, by reducing the mortality by 23% when compared to amiodarone, the Optimal Pharmacological Therapy in Implantable Cardioverter Defibrillator Patients (OPTIC) trial, which represents the largest randomized trial (412 subjects) comparing antiarrhythmic drugs, showed that the use of amiodarone with a beta-blocking agent dramatically reduced shocks from an ICD, by 73%, when compared to the use of sotalol or a beta blocker alone [5,25]. The fact that patients who receive ICD shocks experience reduced quality of life is well known, and this has been a consistent finding in the published studies, that was already evident from data in the first available trials, such as the Antiarrhythmics Versus ICDs (AVID) trial [26]. This is another reason why we chose to administer amiodarone to an increased number of patients in our study. As a result of the above mentioned, the rate of ICD therapies (ATP and shocks) was lower in the present analysis when compared to older studies.

Another major difference to the majority of the studies is the fact that almost all analyses compared NICMP patients receiving ICD with NICMP patients receiving standard medical therapy, instead of comparing NICMP patients to ICMP patients with ICD implant. Exceptions comparing ischemic and non-ischemic heart disease patients treated with primary prophylactic ICDs (Smith et al.) found similar rates with respect to mortality and appropriate ICD shocks at 30 months of follow-up [27]. Rusnak et al., in his 387 consecutive ICD recipients study showed that NICMP was associated with even higher rates of recurrent VT/VF and appropriate ICD therapies compared to ICMP at one year of follow-up, whereas the rates of rehospitalization and all-cause mortality were comparable [28]. These findings were confirmed in a retrospective analysis by Verhagen et al., which also included only patients with ICD for primary prevention, with a median LVEF of 24%; within 40 months of follow-up, the rates of appropriate ICD therapies due to sustained VT/VF and mortality rates were comparable in ICMP and NICMP [29]. Considering these data, we wonder, had the DANISH study also included a comparison between IMCP and NICMP patients receiving ICD, could it still firmly state that ICD does not have a vital role in non-ischemic heart failure with reduced ejection fraction? Meta-analyses including data from all studies over the past 20 years in ICD primary prevention, including the DANISH trial, have confirmed a significant reduction of all-cause mortality associated with ICD use in patients with NICMP [30,31]. This might be a confirmation that the DANISH trial was not sufficiently powered to test its “death from any cause” primary end point over a long follow-up period. With the exception of the inclusion of the DANISH trial, these meta-analyses mainly included trials performed more than one decade ago, and thus mainly reflect older heart failure treatment options. We believe that one of the advantages of our study derives from the maximal optimal medical therapy that the majority of the patients were receiving, thus generalizing the study’s results to the actual modern treatment of heart failure with reduced ejection fraction.

## 5. Limitations

One limitation of the study was the medium follow-up period of 36 months. Patients were included in the period between January 2017 and January 2021, so follow-up of the last included patients was only 7 months, while some patients had a follow-up of up to 55 months. Within this period of time, new heart failure medications have become available, but the device-based therapy criteria have remained the same. This may have affected the outcome through the heterogenicity caused in the study population, but this limitation did not influence the aim of the study, since this limitation applies to both ICMP and NICMP patients.

The present results need to be confirmed within larger and more representative multi-center registry data.

## 6. Conclusions

The present study shows that the rate of cardiovascular death, death from any cause, and sudden cardiac death and the occurrence of ICD-related therapies (appropriate and inappropriate ICD interventions), as well as the recurrences of ventricular tachyarrhythmias in a “real-world” population, are similar after primary prophylactic ICD implantation for both ischemic and non-ischemic cardiomyopathy patients.

## Figures and Tables

**Table 1:** Baseline characteristic of patients with ICD *.

Characteristic	NICMP Group (*n* = 166)	ICMP Group (*n* = 237)
Median age—years	66 (19–81)	65 (35–83)
Male sex, *n* (%)	119 (72)	161 (68)
Median NT-proBNP level—pg/mL	1698 (498–2705)	1765 (399–2967)
Median left ventricular ejection fraction, %	26 +/− 10	24 +/− 10
Median estimated GFR—mL/min/1.73	55 (12–96)	59 (15–98)
NSVT (%)	75 (45)	114 (48)
Medication, *n* (%)		
Amiodarone	55 (33)	97 (41)
ACE I/ARB	138 (83)	194 (82)
Beta-blocker	151 (91)	228 (96)
Loop-diuretics	159 (96)	230 (97)
Mineralocorticoid-receptor antagonist	158 (95)	228 (96)
ARNI	25 (15)	38 (16)
Dapagliflozin	13 (8)	16 (7)
Coexisting conditions, *n* (%)		
Hypertension	106 (64)	161 (68)
Permanent atrial fibrillation	42 (25)	66 (28)
Smoker, *n* (%)	32 (19)	59 (25)
Diabetes mellitus, *n* (%)	23 (14)	43 (18)
Dyslipidemia, *n* (%)	115 (69)	187 (79)
CRT-D patients (%)	32 (19)	40 (17)

* There were no significant differences (*p* > 0.05). NICMP = Non-ischemic cardiomyopathy; ICMP = Ischemic cardiomyopathy; ACE-I = Angiotensin-converting enzyme; ARB = Angiotensin-receptor blocker; GFR = Glomerular filtration rate; NT-pro BNP = N-terminal pro-brain natriuretic peptide; NSVT = Non-sustained ventricular tachycardia (defined as more than 3 consecutive beats originating below the AV node); ARNI = Angiotensin receptor–neprilysin inhibitor.

**Table 2:** Outcomes and adverse events.

Outcome	NICMP Group	ICMP Group	HR (95% CI)	*p* Value
	*n* of patients/total *n* (%)			
Death from any cause	14 (8.4)	18 (7.6)	1.1 (95%CI 0.54–2.21)	0.78
Cardiovascular death	9 (5.4)	14 (5.9)	1 (95% CI 0.45–2.28)	0.97
Sudden cardiac death	2 (1.2)	4 (1.7)	0.71 (95%CI 0.13–3.88)	0.69
Other cardiovascular death	7 (4.2)	10 (4.2)	1.1 (95%CI 0.44–2.87)	0.79
Non-cardiovascular death	4 (2.4)	3 (1.26)	1.2 (95% CI 0.24–2.98)	0.55
Non-sudden deaths	11 (6.6)	13 (5.4)	1 (95% CI 0.41–2.62)	0.67
Appropriate shock or ATP (%)	16 (9.6)	24 (10.1)	1.2 (95%CI 0.62–2.03)	0.8
Inappropriate shock or ATP (%)	7 (4.2)	7 (3)	0.72 (95%CI 0.42–2.93)	0.52
Sustained VT requiring medical intervention/electrical conversion	2 (1.2)	1 (0.42)	1.3(95%CI 0.1–4.01)	0.74
NSVT/NSVF	130 (78.3)	192 (81)	0.68 (95%CI 0.94–1.1)	0.9
			OR (95%CI)	
Device infection *	2 (1.2)	2 (0.84)	1.2 (95%CI 0.12–3.96)	0.48

* Device infection requiring lead extraction or causing death. NICMP = Non-ischemic cardiomyopathy; ICMP = Ischemic cardiomyopathy; VT = Ventricular tachycardia; NSVT = Non-sustained ventricular tachycardia (defined as more than 3 consecutive beats originating below the AV node); ATP = Anti-tachycardia pacing. The cumulative survival plots were estimated according to the Kaplan–Meier method. Survival in groups was compared with the log rank test. Results are reported as the adjusted hazard ratio (HR) with 95% confidence interval (CI). A two-sided *p*-value 0.05 was considered statistically significant. Statistical analyses were performed using the Survival R package.

## Data Availability

The data presented in this study are available on request from the corresponding author.
